# Adverse events affecting recovery from seasonal influenza vaccination in the hypertensive population: A population-based pharmacovigilance analysis

**DOI:** 10.1371/journal.pone.0310474

**Published:** 2025-05-20

**Authors:** Hao Wu, Xiaona He, Yu Cao, Wei Gao

**Affiliations:** 1 Department of Epidemiology and Health Statistics, School of Public Health, Jiangxi Medical College, Nanchang University, Nanchang, Jiangxi, China; 2 Jiangxi Provincial Key Laboratory of Disease Prevention and Public Health, Nanchang University, Nanchang, Jiangxi, China; Regional Health Care and Social Agency of Lodi, ITALY

## Abstract

Seasonal influenza vaccination is crucial for preventing influenza and its complications. Data from the United States Vaccine Adverse Event Reporting System (VAERS) indicate a higher proportion of adverse events (AEs) after influenza vaccination in hypertensive people. However, there is limited evidence on AEs in hypertensive people following seasonal influenza vaccination. We identified 4647 individuals aged 18 years or older with a history of hypertension who received seasonal influenza vaccination and 6380 seasonal influenza-vaccine-induced AEs between 1 January 2013 and 23 June 2023 from VAERS. We identified two groups for comparison: recovery and no recovery from seasonal influenza-vaccine-induced AEs. Propensity score matching (PSM) was performed to adjust for potential confounding factors, including demographic characteristics (age, sex, and region) and season of onset. Cox regression analysis was used to calculate the risk ratio of reported adverse events (AEs) that affected recovery after seasonal influenza vaccination. Most AEs were nonserious and occurred within 48 hours. The most common AEs were general disorders and administration site conditions (therapeutic and non-therapeutic responses, inflammation) and musculoskeletal and connective tissue disorders (musculoskeletal and connective tissue pain and discomfort, bursal disorders, joint-related signs, and symptoms). All three types of seasonal influenza vaccines were associated with injection site reactions (47.07% trivalent influenza vaccine [TIA], hazard ratio, HR 2.04, 95% confidence interval, CI 1.22–3.40; 20.00% quadrivalent influenza vaccine [QIA], HR 2.81, 95% CI, 1.81–4.37; 67.48% influenza vaccine, unknown manufacturer [FLUX], HR 2.83, 95% CI, 1.12–7.15) and were the AEs affecting the largest proportion of delayed recoveries in the hypertensive population. Potential AEs following seasonal influenza vaccination may affect the recovery of the hypertensive population. The majority of AEs reported were general disorders, predominantly injection site reactions, and nonserious.

## Introduction

Influenza is a highly transmissible viral infection caused by the influenza virus, leading to severe respiratory illnesses in approximately 3–5 million people and 290,000–650,000 deaths globally every year [1,2]. Influenza has become a serious public health issue. Since the 20th century, several influenza pandemics have occurred in human history, such as the ‘Spanish Flu’ in 1918–1919, the ‘Asian Flu’ in 1957–1958, the ‘Hong Kong Flu’ in 1968–1969, the ‘Swine Flu’ in 1976, the ‘Russian Flu’ in 1977, and the Influenza A in 2009, which also caused millions of deaths [3,4]. Pandemic and seasonal influenza cause substantial morbidity and mortality worldwide, posing a serious threat to public health and the global economy [5]. Seasonal influenza infections occur in all age groups, but children under 59 months of age, adults over 65 years of age, and pregnant women are more vulnerable [6–8]. Despite the high incidence of annual seasonal influenza infections, annual vaccination programs remain a critical public health tool to reduce the disease burden of seasonal influenza [9]. Currently, seasonal influenza vaccines are produced as ‘trivalent’ or ‘quadrivalent’ formulations [10,11]. Seasonal influenza vaccines are generally considered safe; however, they can sometimes cause adverse events following immunization (AEFIs). Generally, AEFI is deemed less serious than influenza [12]. Therefore, pharmacovigilance (PV) is an important tool for monitoring AEFI and confirming the benefits of immunization in different target groups [13].

The U.S. Vaccine Adverse Event Reporting System (VAERS) was created in 1990 and co-administered by the U.S. Centers for Disease Control and Prevention (CDC) and the U.S. Food and Drug Administration (FDA) to collect spontaneous reports of adverse events (AEs) and manage vaccine safety VAERS accepts reports of AEs from health-care providers, vaccine manufacturers, and the public [14,15]. The main objectives of VAERS are to detect new, unusual, or rare vaccine AEs; assess the safety of newly approved vaccines; make new recommendations for existing vaccines; identify potential risk factors, monitor the increase in known AEs; and determine and address possible reporting clusters [16]. Real-world monitoring of vaccine safety is crucial for understanding suspected AEFIs, assessing their incidence, and determining risk factors to identify potential vaccine contraindications [17]. Moreover, the recently published World Health Organization (WHO) guidelines on the economic evaluation of influenza vaccination suggest considering AEFI when possible [18].

Although inﬂuenza is mostly self-limiting, serious complications can occur in vulnerable patient groups [12]. Hypertension is an established cardiovascular risk factor for the general population. Patients with hypertension are regarded as a high-risk group for being infected with seasonal influenza due to the high possibility of a compromised immune system, especially in the elderly [19,20]. The WHO [1] and U.S. CDC [21] recommend that patients with chronic diseases should be priority groups for influenza vaccination. The Chinese CDC has recommended that it is of vital importance for older hypertensive patients to receive an influenza vaccine [22], which is consistent with the recommendations of the U.S. CDC [23] and the WHO. One study showed that the coverage rate of influenza vaccinations in which only 0.4% of people with chronic diseases had received an influenza vaccine [24], a history of influenza vaccination, and the perceived safety and effectiveness of the vaccination are responsible for the lower values of influenza vaccination coverage in mainland China [25,26]. Based on the existing evidence and knowledge of hypertension and immune response [27,28], potential mechanisms that could explain the observed association between hypertension and increased AEs. Hypertension-induced chronic inflammation could lead to a heightened immune response to the vaccine, resulting in more frequent or severe AEs. Vascular dysfunction, a hallmark of hypertension, could impair the delivery of the vaccine to target tissues, potentially leading to localized reactions at the injection site, such as injection site pain or swelling.

Seasonal influenza vaccination is crucial for preventing influenza and its complications. In addition to influenza vaccination, COVID-19 vaccination has also attracted much attention. Recent studies have found that COVID-19 vaccination may be associated with the onset of hypertension and may increase the risk of death in patients with hypertension [29]. Hypertensive patients are more vulnerable to develop serious complications of COVID-19 [30]. Although the mechanism for hypertension-associated COVID-19 risk remains unclear, prior studies have identified delayed SARS-CoV-2 viral clearance and prolonged inflammatory response among hypertensive patients, which may contribute to greater disease severity. These findings have raised concerns about the safety of vaccination, especially for high-risk groups such as hypertensive patients.

With the aim of providing further evidence on possible vaccine-related adverse reactions, to examine various seasonal influenza-vaccine-induced AEs in the hypertensive population, we collected comprehensive data comprised three different file from three seasonal influenza vaccines (trivalent influenza vaccine [TIA], quadrivalent influenza vaccine [QIA], and influenza vaccine, unknown manufacturer [FLUX]), reported to the VAERS from 1 January 2013–23 June 2023. AEs were examined in the propensity-score-matched populations, adjusted for demographic characteristics (age, sex and region) and season of onset. Major AEs and their adjusted hazard ratios were estimated using a Cox proportional hazards regression model.

## Materials and methods

### Study data selection

This was a retrospective observational study utilizing data from the United States Vaccine Adverse Event Reporting System (VAERS) database. We collected data on individuals aged 18 years or older with a history of hypertension who received seasonal influenza vaccination from 1 January 2013–23 June 2023. A total of 81,713 AEs were initially investigated during the study period; of these, 4647 individuals with a history of hypertension with a unique VAERS ID were screened: 2135 for TIA, 2300 for QIA, and 212 for FLUX. Based on VAERS data including recovery information, we categorized subjects into two groups: those who recovered within one month of the AEs and those who did not. The primary endpoint was the occurrence of adverse events within one month after seasonal influenza vaccination in hypertensive individuals, including those with controlled and uncontrolled hypertension. The exclusion criteria were missing data on age or being under 18 years of age, missing data on the number of days associated with vaccine-related AEs, cases reported after one month, failure to report recovery from vaccine-induced AEs, outliers or flawed logic, and data for which the year of vaccination did not coincide with the year reported. S1 Fig in S1 Appendix displays the workflow, illustrating the data collection process and the number of individuals excluded at different stages.

### Study population

Data from the original reports are publicly available as an online downloadable dataset on the VAERS website, with sensitive patient information removed [14]. The raw dataset comprised three distinct files in the VAERS database. It included demographic information and medical history in VAERSDATA, vaccination-associated adverse symptoms in VAERSSYMPTOMS, and vaccine type in VAERSVAX. We identified all cases related to the seasonal influenza vaccine by using a unique case identification number in the raw dataset. We examined the medical records for each individual’s medical history, particularly focusing on pre-diagnosed diseases at the time of seasonal vaccination. We used keyword search to identify hypertensive patients from the VAERS database, such as high blood pressure, hypertension, HBP, HTN, and blood pressure to extract individuals with a history of high blood pressure as the primary population. The analysis of medical records included general information (VAERS ID, age, sex, region, and medical history when vaccinated), vaccination information (vaccine type, vaccination date, AEs onset date, and the interval between the vaccination date and the occurrence of AEs), and recovery from vaccine-induced AEs.

### Selection of AE reports

According to the unique ID identification number, 4647 individuals with a history of hypertension were included after the deletion of blank values, age under 18 years, and certain records with logical errors. All adverse events after the seasonal influenza vaccination are documented in VAERSSYMPTOMS, and we extracted all valid adverse symptoms. Additionally, we examined five adverse symptoms for each participant to more precisely measure the AEs affecting their recovery from seasonal influenza vaccination. Each case had a maximum of five terms, with each term listed for the corresponding VAERS ID. We have combined multiple VAERS IDs into the same patient ID. Seasonal influenza vaccine AEs were defined as reports submitted that contained the word ‘FLU’, but did not specifically mention H1N1 influenza in the vaccine code. To compare the different types of seasonal influenza vaccines, we classified them into three primary categories: (1) TIA, including FLU3, FLUA3, FLUC3, FLUR3, and FLUN3; (2) QIA, including FLU4, FLUA4, FLUC4, FLUR4, and FLUN4; and (3) FLUX with no brand name.

### Standard medical terms for vaccine AEs

The Medical Dictionary for Regulatory Activities (MedDRA) is a medical coding dictionary used by regulatory authorities, pharmaceutical companies, clinical research organizations, and health care professionals [31]. MedDRA is organized into a five-level hierarchy, with the highest level being the Systematic Organ Classification (SOC), which is further divided into High-Level Group Terms (HLGT), High-Level Terms (HLT), Preferred Terms (PT), and Low-Level Terms (LLT). In release version 26.1 [32], the MedDRA hierarchy consists of 27 SOC, 354 HLGT, 1,855 HLT, 26,180 PT, and 87,592 LLT terms. AEs in the VAERS database are coded using the MedDRA terminology. The VAERS database provides several AEs at the PT level, but these are highly fragmented into signs, symptoms, diagnoses, investigations, or medical procedures, which may fail to recognize differences in the incidence of AEs [33]. Therefore, we converted the standard terminology associated with all AEs (PT) into HLT [34]. HLT terms were coded by two trained coders and a coding consistency test was conducted, with a Kappa coefficient of 0.85. We also performed a series of data cleaning and preprocessing steps, including handling missing values, outliers, and duplicate records. AEs at the primary SOC level were selected for MedDRA’s hierarchical structure analysis. Each AE encoded a binary value indicating whether the seasonal influenza vaccine recipient had certain adverse symptoms, where 1 indicated ‘YES’ and 0 indicated ‘NO’ [34].

### Statistical analysis

The VAERS database collected data on age groups, sex, region, and season of onset. The type of vaccine used may depend on individual characteristics; therefore, individual choices may influence vaccine-associated AEs and recovery. Therefore, we applied propensity score matching (PSM) to reduce bias due to these confounding variables between two groups (recovery and no recovery from seasonal influenza- vaccine-induced AEs). A nearest-neighbor approach with a 1:1 matching ratio was used. PSM shifts the regulation of confounders to the control of propensity scores to ‘downscale’ and to control for confounding bias. The propensity score (PS) for seasonal influenza vaccine exposure was estimated using logistic regression modeling with age group (18–59, 60–64, 65–69, 70–74, 75–79, and ≥ 80 years), region (West, Midwest, Northeast, South, and Overseas territory), sex (female and male), and season of onset (spring including March, April, and May; summer including June, July, and August; fall including September, October, and November; and winter including December, January, and February) as predictors. To control for confounding variables, estimates were obtained using a binomial logistic regression analysis. The caliper value was defined as ‘0.2×standard deviation of the PS estimates with logit transformation applied’. Standardized mean difference (SMD) was calculated to assess variable balance in the matched data, and a SMD value <0.1 was considered balanced [35].The case selection for proportional imbalance analysis is shown in S1 Fig in S1 Appendix.

The Kaplan-Meier curves were used to estimate the incidence of non-recovery in the hypertensive population following the three types of influenza vaccines within a month. S2 Fig in S1 Appendix shows the cumulative incidence of non-recovery in the hypertensive population for each vaccine type. A total of 6380 seasonal influenza vaccine-induced AEs reported for each individual over one month were considered independent variables in our study. The dependent variable was whether the hypertensive population recovered from influenza vaccine-induced AEs or not. Due to the annual changes in influenza vaccine formulations, considering the potential impact of these changes on the study results, therefore, we also considered potential confounding factors such as the timing of vaccination, number of doses, route of administration, history of allergies, and previous vaccination history, and made adjustments in the model. Cox proportional hazards regression analysis was used to examine the association between these AEs and recovery. AEs with a total frequency of less than 10 were removed to address any monotonic likelihood issues that may have occurred in the Cox proportional hazards regression models [34]. The results of the Cox regression models are expressed as hazard ratios (HR) and 95% confidence intervals (CI). A consistency index was used to determine the discriminatory power of the multivariate Cox proportional hazard regression model. All statistical analyses were performed using R version 4.3.2 (R Foundation for Statistical Computing, Vienna, Austria). Prism version 9 (GraphPad Software, USA) was used as a mapping tool, and the MedDRA desktop viewer was used for hierarchical structure analysis of the medical terms [33,34].

## Results

Of the 4,647 validated cases with a history of hypertension, the mean baseline age of the study participants was 64.46 years. The gender distribution was 3,132 females (67.40%), and 1,500 males (32.28%), with a male-to-female ratio of approximately 1:2.08. The southern region had the highest number of seasonally influenza-vaccinated hypertensive individuals (34.02%). In contrast, there was little difference in the number of individuals vaccinated in the western (22.04%) and midwestern regions (23.28%). The AE period coincided with the peak season for seasonal influenza vaccination in autumn, which had the highest number of episodes among the four seasons. Additionally, the AEs episode interval for the seasonal influenza vaccine was 3.30 days: 3.03 for TIA; 3.38 for QIA; and 5.06 for FLUX. The results showed that 2,188 (47.08%) patients in the hypertensive population did not recover from AEs (Table 1). After PSM was paired at 1:1 ratio to eliminate selection bias, the caliper width was adjusted to 0.2, resulting in the disappearance of differences between groups (SMD < 0.1, P > 0.05). The total number of seasonal influenza vaccinations after matching was 3,914. We found differences in age, sex, region, and season of onset between the non-recovered and recovered groups in the hypertensive population before PSM for TIA, QIA, and FLUX (S1–S3 Tables in S1 Appendix).

**Table 1:** Demographic characteristics of seasonal influenza vaccination in the hypertensive population.

Variable		TIA [*N* (%)]	QIA [*N* (%)]	FLUX [*N* (%)]	Total [*N* (%)]
Total		2,135(45.95)	2,300(49.49)	212(4.56)	4,647(100.00)
Age [mean (SD)]		68.33 ± 11.51	61.35 ± 13.09	59.28 ± 13.86	64.46 ± 12.94
Age group(years)	18-59	378 (17.70)	938 (40.78)	102 (48.11)	1,418(30.51)
	60-64	137 (6.42)	395 (17.17)	26 (12.26)	558(12.01)
	65-69	615 (28.81)	379 (16.48)	34 (16.04)	1,028(22.12)
	70-74	401 (18.78)	241 (10.49)	24 (11.32)	666(14.13)
	75-79	289 (13.54)	185 (8.04)	11 (5.19)	485(10.44)
	≥80	315 (14.75)	162 (7.04)	15 (7.08)	492(10.59)
Sex	Female	1,447 (67.78)	1,548 (67.30)	137 (64.62)	3,132(67.40)
	Male	679 (31.80)	746 (32.44)	75 (35.38)	1,500(32.28)
	Unknown	9 (0.42)	6 (0.26)	0 (0.00)	15(0.32)
Region	West	495 (23.19)	469 (20.39)	60 (28.30)	1,024(22.04)
	Midwest	460 (21.55)	576 (25.04)	46 (21.70)	1,082(23.28)
	Northeast	434 (20.33)	399 (17.35)	37 (17.45)	870(18.72)
	South	712 (33.35)	807 (35.09)	62 (29.25)	1,581(34.02)
	Overseas territory	34 (1.58)	49 (2.13)	7 (3.30)	90(1.94)
Onset season	Spring	224 (10.49)	250 (10.87)	14 (6.60)	488(10.50)
	Summer	308 (14.43)	303 (13.17)	28 (13.21)	639(13.75)
	Autumn	1,271 (59.53)	1,375 (59.79)	137 (64.62)	2,783(59.89)
	Winter	332 (15.55)	372 (16.17)	33 (15.57)	737(15.86)
Recovery	Yes	1,263 (59.16)	1,096 (47.65)	100 (47.17)	2,459(52.92)
	No	872 (40.84)	1,204 (52.35)	112 (52.83)	2,188(47.08)
AEs onset interval(days) [mean (SD)]		3.03 ± 27.46	3.38 ± 21.90	5.06 ± 27.52	3.30 ± 24.86
AEs median onset interval(range) in days		0(0–1097)	0 (0–731)	1 (0–359)	0 (0–1097)

SD, standard deviation; TIA, trivalent influenza vaccine; QIA, quadrivalent influenza vaccine; FLUX, influenza vaccine, unknown manufacturer; AEs, adverse events.

**Fig 1:**
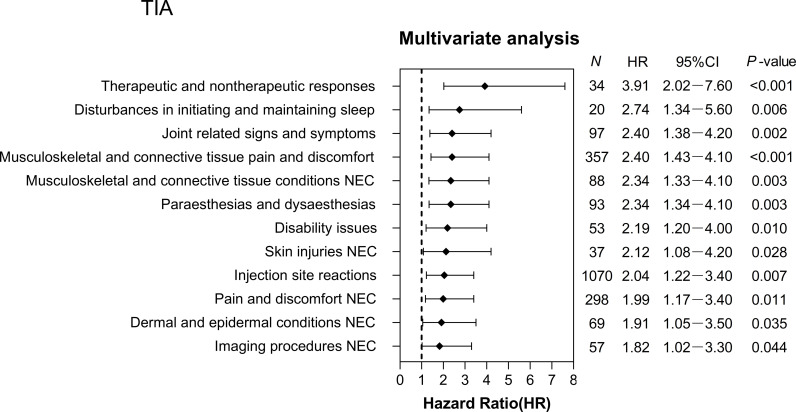
Adverse events (AEs) hazard ratios after trivalent influenza vaccines (TIA) vaccination estimated from a multivariate Cox proportional hazard model. The results showed 12 trivalent influenza vaccine (TIA) vaccine‐induced adverse events (AEs) with a statistically significant difference (*P* < 0.05), estimated using multivariate Cox proportional hazard regression analysis. N denotes the number of adverse cases in the non-recovered group; HR, hazard ratio; CI, confidence interval.

**Fig 2:**
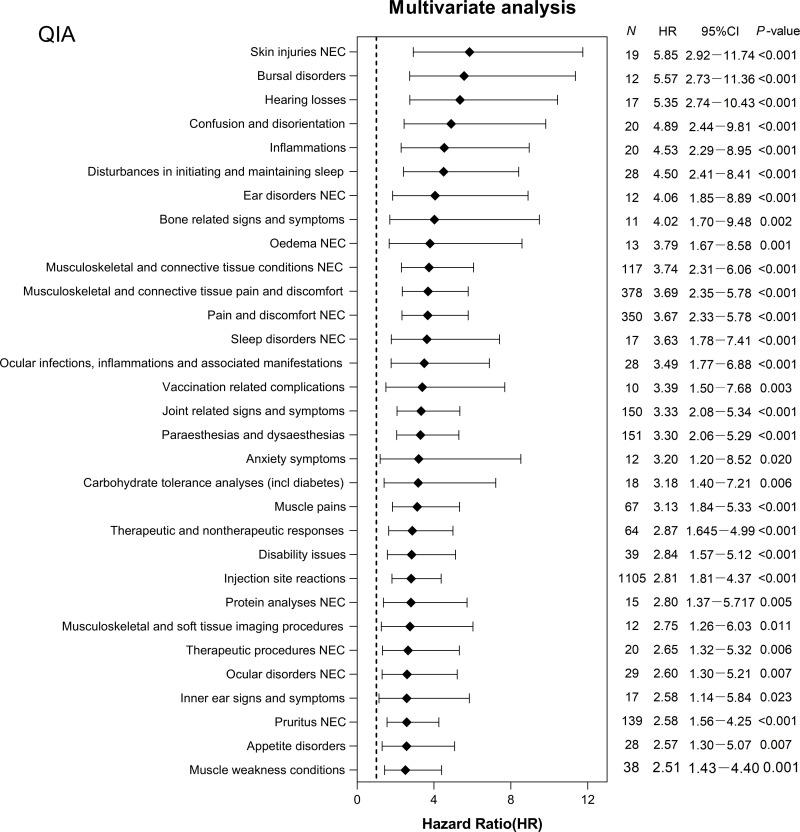
Adverse events (AEs) hazard ratios after quadrivalent influenza vaccines (QIA) vaccination estimated from a multivariate Cox proportional hazard model. The results showed that 57 quadrivalent influenza vaccine (QIA) vaccine‐induced adverse events (AEs) with a statistically significant difference (*P* < 0.05), estimated with a multivariate Cox proportional hazard regression analysis. N denotes the number of adverse cases in the non-recovered group; HR, hazard ratio; CI, confidence interval.

The Kaplan-Meier survival curves indicated that, among the three types of influenza vaccines, the hypertensive population had the lowest non-recovery incidence within one month following the TIA vaccine (S2 Fig in S1 Appendix). In the PS-matched hypertensive population, 75 AEs with TIA have been reported in the PS-matched hypertensive population (S4 Table in S1 Appendix). Furthermore, we identified 12 AEs affecting recovery that were associated with the estimated risk of occurrence using a multivariate Cox regression model with a consistency index of 0.58. Overall, 12 AEs were linked to TIA (Fig 1), with an adjusted HR range of 1.82 to 3.91, and were categorized into eight SOC terms (S5 Table in S1 Appendix). Among the SOC terms, two common AEs accounted for the majority: general disorders and administration site conditions (61.68%) and musculoskeletal and connective tissue disorders (23.85%). Especially, injection site reactions (47.07%; HR = 2.04; 95% CI, 1.22–3.40), musculoskeletal and connective tissue pain and discomfort (15.71%; HR = 2.40; 95% CI, 1.43–4.10), pain and discomfort NEC (13.11%; HR = 1.99; 95% CI, 1.17–3.40) showed an increased risk of non-recovery in hypertensive individuals. After the QIA, 94 AEs were reported (S6 Table in S1 Appendix), and 57 AEs were estimated using the multivariate Cox regression model, with a consistency index of 0.63 (Fig 2). The adjusted HR distribution ranged from 2.08 to 5.85 and was grouped into 15 SOC terms (S7 Table in S1 Appendix).

**Fig 3:**
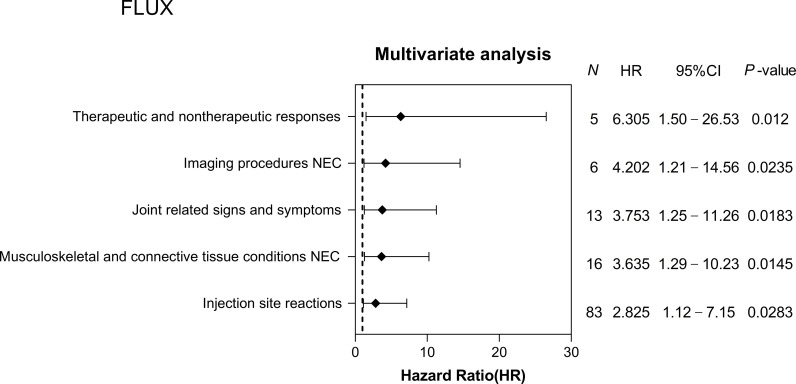
Adverse events (AEs) hazard ratios after influenza unknown manufacturer (FLUX) vaccination estimated from a multivariate Cox proportional hazard model. The results showed five influenza unknown manufacturer (FLUX) vaccine-induced adverse events (AEs) with a statistically significant difference (*P* < 0.05), estimated using multivariate Cox proportional hazard regression analysis. N denotes the number of adverse cases in the non-recovered group; HR, hazard ratio; CI, confidence interval.

Four AEs were more common in the non-recovery group: general disorders and administration site conditions (46.74%), musculoskeletal and connective tissue disorders (14.95%), skin and subcutaneous tissue disorders (12.17%), and nervous system disorders (9.63%). Among them, injection site reactions (20.00%; HR = 2.81; 95% CI, 1.81–4.37), musculoskeletal and connective tissue pain and discomfort (6.84%; HR = 3.69; 95% CI, 2.35–5.78), pain and discomfort NEC (6.34%; HR = 3.67; 95% CI, 2.33–5.78), asthenic conditions (5.39%; HR = 2.25; 95% CI, 1.41–3.58) typically exhibited a higher risk with QIA. Additionally, 28 AEs with FLUX were observed (S8 Table in S1 Appendix) and five AEs were associated with the risk of developing AE, as estimated by the multivariate Cox regression model (Fig 3). The consistency index is 0.59. The adjusted HR distribution ranged from 2.83 to 6.31 and was subsequently categorized into three SOC terms (S9 Table in S1 Appendix). General disorders, administration site conditions (71.54%), and musculoskeletal and connective tissue disorders (23.58%) were the most frequent (similar to the TIA results). In particular, injection site reactions (67.48%; HR = 2.83; 95% CI, 1.12–7.15), tissue condition NEC (13.01%; HR = 3.64; 95% CI, 1.29–10.23), and joint-related signs and symptoms (10.57%; HR = 3.75; 95% CI, 1.25–11.26) were at heightened risk in the non-recovery group. A comparison of PSM before and after TIA, QIA, and FLUX vaccinations is shown in S3 Fig in S1 Appendix.

## Discussion

We analyzed whether major AEs influenced recovery following seasonal influenza vaccination in a hypertensive population. Among the 27 SOCs examined, four terms (general disorders and administration site conditions, musculoskeletal and connective tissue disorders, skin and subcutaneous tissue disorders, and nervous system disorders) were associated with a higher frequency of AEs in the PS-matched hypertensive population. Among the non-recovery group, the most common AEs were injection site reactions, musculoskeletal and connective tissue pain and discomfort, pain and discomfort NEC, and joint-related signs and symptoms. Most AEs (95.5%) occurred within 48 hours of vaccination. These results are consistent with those of several previous studies, suggesting that local and systemic reactions are common [36,37], and that all reported AEs were nonserious. Furthermore, our study showed results related to neurological aspects after seasonal influenza vaccination in the hypertensive population. Paraesthesias and dysesthesias (4.09%) were the main manifestations of TIA, and nervous system disorders were found in 9.63% with QIA; the most frequent AEs were headache NEC (3.33%), neurological signs and symptoms NEC (2.55%), paresthesia, and dysesthesia (2.73%). In addition, no association was found between FLUX and neurological aspects. Some studies have shown an association between the incidence of Greene-Barrow syndrome [37], Acute Disseminated Encephalomyelitis (ADEM), and seasonal influenza vaccination [38].

After TIA vaccination, there were 20 cases (0.88%) of disturbances in initiating and maintaining sleep, and 37 cases (1.63%) of NEC skin injuries in the population. Disturbances in initiating and maintaining sleep are psychiatric disorders; however, no studies have been conducted on seasonal influenza vaccines and psychiatric disorders. In a study evaluating the relationship between maternally inactivated influenza vaccination (IIV) and the risk of diagnosis of neurodevelopmental disorders in early childhood, no increased risk of neurodevelopmental disorders was found in children after maternal exposure to IIV [39]. The majority of rare AEs associated with QIA, including ear and labyrinth disorders (0.83%), eye disorders (1.03%), appetite disorders (0.51%), and respiratory, thoracic, and mediastinal disorders (0.83%), may hinder recovery in the hypertensive population. Rare AEs associated with vaccination with FLUX were primarily NEC (4.88%) in the hypertensive population.

While the Cox regression models demonstrated moderate discriminatory ability, with consistency indices ranging from 0.58 to 0.63 across vaccine types, our values suggest clinically meaningful predictive capacity for identifying AEs hindering recovery in hypertensive populations. This moderate performance aligns with the inherent complexity of post-vaccination recovery processes, which are influenced by sample heterogeneity and unmeasured confounders. To enhance model precision, the model can be optimized by using multicenter data, such as the SEER database, or by integrating active surveillance systems like electronic health records. Moreover, future studies should incorporate biomarkers of chronic inflammation (e.g., CRP, IL-6) and vascular compliance metrics, as recent pathophysiological models suggest that these factors may account for 38–42% of the unexplained variance in AEs severity [27].

Injection site reactions (TIV: 47.07%, HR = 2.04; QIV: 20.00%, HR = 2.81; FLUX: 67.48%, HR = 2.83) were the most common AEs reported and significantly associated with delayed recovery, highlighting the need for pre-vaccination counseling on managing localized inflammation. These reactions were the predominant barrier to recovery across all three vaccine types, emphasizing the importance of individualized monitoring and follow-up care for hypertensive individuals after vaccination. Musculoskeletal AEs also demonstrated vaccine-type specificity, particularly with QIV, where pain disorders (HR = 3.69, 95%CI 2.35–5.78) necessitated targeted NSAID prophylaxis in hypertensive individuals [40]. Additionally, delayed neurological manifestations emerged as a concern, with a median onset of 7 days post-vaccination and accounting for 9.63% of non-recovery cases. This finding underscores the need for extended monitoring in patients with pre-existing neurovascular comorbidities. While the majority of AEs were non-serious and resolved within a short period, the presence of persistent symptoms in some individuals emphasizes the importance of individualized monitoring and follow-up care for hypertensive individuals after vaccination.

Our study observed differences in the incidence of adverse events (AEs) among three types of influenza vaccines (TIV, QIV, and FLUX), with TIV associated with the lowest incidence, necessitate further research to explore the underlying reasons and identify the most appropriate vaccine options for hypertensive individuals. Future vaccine formulations should prioritize antigen-adjuvant combinations that minimize localized inflammatory responses without compromising immunogenicity [41]. Influenza viruses are highly variable, with their genetic and antigenic characteristics changing each year. To address this variability, influenza vaccine formulations are adjusted annually to include the circulating influenza virus strains for that year based on recommendations from the WHO [1]. Given the inherent variability of influenza viruses and the annual adjustments made to vaccine formulations, it is crucial to investigate the impact of these changes on vaccine safety, particularly in hypertensive individuals.

Clinical trials are the gold standard to evaluate the safety and efficacy of new vaccines. However, they have limitations in evaluating rare AEs due to the short observation period and limited size of the testing populations. Post-marketing surveillance, such as a spontaneous reporting database, plays a critical role in continually monitoring the safety signals of AEs [42]. Nevertheless, our study had some limitations. First, the VAERS database suffers from underreporting, overreporting, missing data, and possible reporting based on indirect information such as media sources [36]. The FDA estimates that fewer than 1% of adverse events are reported via VAERS [43], indicating a significant underreporting bias. This suggests that the observed rates of serious AEs in our study may be underestimated, potentially leading to an overestimation of the safety of seasonal influenza vaccines in hypertensive individuals. VAERS relies on voluntary reporting from healthcare providers, vaccine manufacturers, and the public, which can result in incomplete reports. Moreover, we cannot rule out the possibility of conditions or regulatory agency interventions due to unadjusted confounders, such as exposure to previous infections and individual occupational characteristics [44,45], which may affect the VAERS database information based on the year of reporting. Therefore, our findings may have involved unknown or invalidated AEs. Furthermore, the reduced sample size of the post-PSM study may have led to a selection bias, and we used a limited number of covariates that met the user-defined sample thresholds in the propensity score-matched target outcome. Our results showed that the average time of adverse events reporting was at 3 days after vaccination, which may be too early to capture the final recovery of all patients, suggesting that we may have overestimated the reported non-recovery. At the same time, this bias may lead our study results to be conservative in assessing vaccine safety, as some short-term adverse events may not result in long-term consequences. In future studies, we will focus on how to reduce this bias. In addition, further research needs to consider limiting to only serious adverse events and restricting our analysis to the pre-COVID years, to validate these findings and assess the true impact of AEs on recovery.

## Conclusion

Our study provides valuable insights into the AEs associated with seasonal influenza vaccination in hypertensive individuals, demonstrating the potential impact of common reactions such as injection site reactions and systemic reactions like musculoskeletal pain and discomfort on the recovery process. Importantly, our findings highlight that these AEs, while generally non-serious, may impact the recovery process in hypertensive patients. This information is crucial for healthcare providers to develop tailored post-vaccination monitoring and management strategies in this vulnerable population, ultimately promoting safer and more effective vaccination strategies.

## Supporting information

S1 Appendix Supplementary materials. (DOCX)
