# Supplementary material for: Adverse events affecting recovery from seasonal influenza vaccination in the hypertensive population: A population-based pharmacovigilance analysis
Source: PLoS One. 2025 May 20;20(5):e0310474. doi: 10.1371/journal.pone.0310474 (PMC12091759; doi:10.1371/journal.pone.0310474)

**Supplementary materials**

**Table S1. Baseline characteristics of the** **trivalent influenza vaccines (TIA)-vaccinated hypertensive population before and after** **propensity score matching (PSM).**

| **Variable** | | **Before propensity score matching** | | | | **After propensity score matching** | | | |
| --- | --- | --- | --- | --- | --- | --- | --- | --- | --- |
|  |  | **N (%)** | | **SMD** | ***P*-value** | **N (%)** | | **SMD** | ***P*-value** |
|  |  | **Recovery** | **No Recovery** |  |  | **Recovery** | **No Recovery** |  |  |
| Total, *N* | | 1263 | 872 |  |  | 853 | 853 |  |  |
| Age group | 18-59 | 239(18.92) | 139(15.94) | 0.126 | 0.018 | 142(16.65) | 139(16.30) | 0.055 | 0.867 |
|  | 60-64 | 88(6.97) | 49(5.62) |  |  | 52(6.10) | 49(5.74) |  |  |
|  | 65-69 | 358(28.35) | 257(29.47) |  |  | 244(28.60) | 250(29.31) |  |  |
|  | 70-74 | 222(17.58) | 179(20.53) |  |  | 172(20.16) | 174(20.40) |  |  |
|  | 75-79 | 163(12.90) | 126(14.45) |  |  | 120(14.07) | 121(14.18) |  |  |
|  | ≥80 | 193(15.28) | 121(13.99) |  |  | 123(14.42) | 120(14.07) |  |  |
| Sex | Female | 867(68.65) | 580(66.51) | 0.143 | 0.023 | 575(67.41) | 569(66.71) | 0.030 | 0.825 |
|  | Male | 389(30.80) | 290(33.26) |  |  | 277(32.47) | 282(33.06) |  |  |
|  | Unknown | 7(0.55) | 2(0.23) |  |  | 1(0.12) | 2(0.23) |  |  |
| Region | West | 313(24.78) | 182(20.87) | 0.169 | 0.005 | 174(20.40) | 182(21.34) | 0.051 | 0.895 |
|  | Midwest | 277(21.93) | 183(20.99) |  |  | 192(22.51) | 181(21.22) |  |  |
|  | Northeast | 271(21.46) | 163(18.69) |  |  | 152(17.82) | 163(19.11) |  |  |
|  | South | 386(30.56) | 326(37.39) |  |  | 322(37.75) | 314(36.81) |  |  |
|  | Others | 16(1.27) | 18(2.06) |  |  | 13(1.52) | 13(1.52) |  |  |
| Onset season | Spring | 133(10.53) | 91(10.44) | 0.150 | 0.008 | 90(10.55) | 89(10.43) | 0.010 | 0.998 |
|  | Summer | 155(12.27) | 153(17.55) |  |  | 141(16.53) | 137(16.06) |  |  |
|  | Autumn | 774(61.28) | 497(57.00) |  |  | 497(58.26) | 496(58.15) |  |  |
|  | Winter | 201(15.91) | 131(15.02) |  |  | 125(11.23) | 131(15.36) |  |  |
| Age(years) | | 67.96±12.08 | 68.86±10.63 | 0.079 | 0.076 | 68.52±11.23 | 68.74±10.64 | 0.013 | 0.796 |

SMD, standardized mean difference.**Table S2. Baseline characteristics of the quadrivalent influenza vaccines (QIA)- vaccinated hypertensive population before and after propensity score matching (PSM).**

| **Variable** | | **Before propensity score matching** | | | | **After propensity score matching** | | | |
| --- | --- | --- | --- | --- | --- | --- | --- | --- | --- |
|  |  | **N (%)** | | **SMD** | ***P*-value** | **N (%)** | | **SMD** | ***P*-value** |
|  |  | **Recovery** | **No Recovery** |  |  | **Recovery** | **No Recovery** |  |  |
| Total, *N* | | 1096 | 1204 |  |  | 1027 | 1027 |  |  |
| Age group | 18-59 | 423(38.59) | 515(42.77) | 0.154 | 0.009 | 423(41.19) | 436(42.45) | 0.051 | 0.852 |
|  | 60-64 | 184(16.79) | 211(17.52） |  |  | 177(17.23) | 180(17.53) |  |  |
|  | 65-69 | 174(15.88) | 205(17.04） |  |  | 171(16.65) | 150(14.61) |  |  |
|  | 70-74 | 116(10.58) | 125(10.38） |  |  | 107(10.42) | 119(11.59) |  |  |
|  | 75-79 | 104(9.49) | 81(6.73) |  |  | 86(8.37) | 78(7.59) |  |  |
|  | ≥80 | 95(8.67) | 67(5.56) |  |  | 63(6.14) | 64(6.23) |  |  |
| Sex | Female | 734(66.97) | 814(67.61) | 0.014 | 0.944 | 692(67.38) | 672(65.43) | 0.041 | 0.645 |
|  | Male | 359(32.76) | 387(32.14) |  |  | 332(32.33) | 352(34.28) |  |  |
|  | Unknown | 3(0.27) | 3(0.25) |  |  | 3(0.29) | 3(0.29) |  |  |
| Region | West | 243(22.17) | 226(18.77) | 0.127 | 0.045 | 225(21.91) | 220(21.42) | 0.023 | 0.991 |
|  | Midwest | 271(24.73) | 305(25.33) |  |  | 251(24.44) | 261(25.41) |  |  |
|  | Northeast | 189(17.24) | 210(17.44) |  |  | 185(18.01) | 184(17.92) |  |  |
|  | South | 363(33.12) | 444(36.88) |  |  | 348(33.89) | 344(33.50) |  |  |
|  | Others | 30(2.74) | 19(1.58) |  |  | 18(1.75) | 18(1.75) |  |  |
| Onset season | Spring | 124(11.31) | 126(10.46) | 0.130 | 0.021 | 109(10.61) | 122(11.88) | 0.040 | 0.841 |
|  | Summer | 168(15.33) | 135(11.21) |  |  | 137(13.34) | 134(13.05) |  |  |
|  | Autumn | 630(57.48) | 745(61.88) |  |  | 618(60.18) | 609(59.30) |  |  |
|  | Winter | 174(15.88) | 198(16.45) |  |  | 163(15.87) | 162(13.17) |  |  |
| Age(years) | | 62.07±13.47 | 60.69±12.71 | 0.105 | 0.012 | 61.40±13.02 | 60.90±13.17 | 0.038 | 0.388 |

SMD, standardized mean difference.

**Table S3. Baseline characteristics of the** **influenza unknown manufacturer (FLUX) -vaccinated hypertensive population before and after propensity score matching (PSM).**

| **Variable** | | **Before propensity score matching** | | | | **After propensity score matching** | | | |
| --- | --- | --- | --- | --- | --- | --- | --- | --- | --- |
|  |  | **N (%)** | | **SMD** | ***P*-value** | **N (%)** | | **SMD** | ***P*-value** |
|  |  | **Recovery** | **No Recovery** |  |  | **Recovery** | **No Recovery** |  |  |
| Total, *N* | | 100 | 112 |  |  | 77 | 77 |  |  |
| Age group | 18-59 | 47(47.00) | 55(49.11) | 0.173 | 0.814 | 35(45.45) | 35(45.45) | 0.063 | 0.940 |
|  | 60-64 | 12(12.00) | 14(12.50) |  |  | 9(11.69） | 9(11.69) |  |  |
|  | 65-69 | 17(17.00) | 17(15.18） |  |  | 12(15.59) | 14(18.18) |  |  |
|  | 70-74 | 12(12.00) | 12(10.71) |  |  | 11(14.29) | 10(12.99) |  |  |
|  | 75-79 | 6(6.00) | 5(4.46） |  |  | 4(5.19) | 3(3.90) |  |  |
|  | ≥80 | 6(6.00) | 9(8.04) |  |  | 6(7.79) | 6(7.79) |  |  |
| Sex | Female | 67(67.00) | 70(62.50) | 0.084 | 0.494 | 47(61.04) | 52(67.53) | 0.096 | 0.901 |
|  | Male | 33(33.00) | 42(37.50) |  |  | 30(38.96) | 25(32.47) |  |  |
|  | Unknown | 0(0.00) | 0(0.00) |  |  | 0(0.00) | 0(0.00) |  |  |
| Region | West | 31(31.00) | 29(25.89) | 0.340 | 0.205 | 22(28.57) | 22(28.57) | 0.074 | 0.995 |
|  | Midwest | 27(27.00) | 19(16.97) |  |  | 16(20.78) | 18(23.38) |  |  |
|  | Northeast | 16(16.00） | 21(18.75) |  |  | 14(18.18) | 14(18.18) |  |  |
|  | South | 24(24.00) | 38(33.93) |  |  | 23(29.87) | 21(27.27) |  |  |
|  | Others | 2(2.00) | 5(4.46) |  |  | 2(2.60) | 2(2.60) |  |  |
| Onset season | Spring | 10(10.00) | 4(3.57) | 0.430 | 0.025 | 4(5.19) | 4(5.19) | 0.091 | 0.906 |
|  | Summer | 12(12.00) | 16(14.29) |  |  | 11(14.29) | 10(12.99) |  |  |
|  | Autumn | 69(69.00) | 68(60.71) |  |  | 54(70.13) | 52(67.53) |  |  |
|  | Winter | 9(9.00) | 24(21.43) |  |  | 8(10.39) | 11(14.29) |  |  |
| Age(years) | | 59.63±13.82 | 58.97±13.95 | 0.047 | 0.731 | 60.87±13.87 | 59.95±12.83 | 0.069 | 0.669 |

SMD, standardized mean difference.

**Table S4. Adverse events (AEs) with trivalent influenza vaccines (TIA) vaccination in the PS-matched hypertensive population.**

| **SOC** | **HLT** | **Total (%)** | **N (%)** |
| --- | --- | --- | --- |
| Blood and lymphatic system disorders | Lymphatic system disorders NEC | 13(0.24) | 13(0.24) |
| Ear and labyrinth disorders | Ear disorders NEC | 25(0.47) | 13(0.24) |
|  | Hearing losses |  | 12(0.23) |
| Eye disorders | Ocular disorders NEC | 42(0.79) | 15(0.28) |
|  | Ocular infections, inflammations and associated manifestations |  | 27(0.51) |
| Gastrointestinal disorders | Diarrhoea (excl infective) | 280(5.24) | 57(1.07) |
|  | Gastrointestinal and abdominal pains (excl oral and throat) |  | 21(0.39) |
|  | Gastrointestinal signs and symptoms NEC |  | 33(0.62) |
|  | Nausea and vomiting symptoms |  | 130(2.43) |
|  | Oral soft tissue signs and symptoms |  | 18(0.34) |
|  | Oral soft tissue swelling and oedema |  | 21(0.39) |
| General disorders and administration site conditions | Asthenic conditions | 2384(44.63) | 263(4.92) |
|  | Febrile disorders |  | 153(2.86) |
|  | Feelings and sensations NEC |  | 276(5.17) |
|  | Gait disturbances |  | 30(0.56) |
|  | General signs and symptoms NEC |  | 235(4.40) |
|  | Injection site reactions |  | 1070(20.03) |
|  | Pain and discomfort NEC |  | 298(5.58) |
|  | Therapeutic and nontherapeutic responses |  | 34(0.64) |
|  | Vaccination site reactions |  | 25(0.47) |
| Immune system disorders | Allergic conditions NEC | 12(0.23) | 12(0.23) |
| Infections and infestations | Bacterial infections NEC | 65(1.22) | 36(0.68) |
|  | Herpes viral infections |  | 16(0.30) |
|  | Infections NEC |  | 13(0.24) |
| Injury, poisoning and procedural complications | Non-site specific injuries NEC | 78(1.46) | 14(0.26) |
|  | Product administration errors and issues |  | 27(0.51) |
|  | Skin injuries NEC |  | 37(0.69) |
| Investigations | Blood counts NEC | 372(6.96) | 13(0.24) |
|  | Carbohydrate tolerance analyses (incl diabetes) |  | 16(0.30) |
|  | Cardiac function diagnostic procedures |  | 10(0.19) |
|  | Central nervous system imaging procedures |  | 22(0.41) |
|  | Cerebrospinal fluid tests (excl microbiology) |  | 10(0.19) |
|  | ECG investigations |  | 19(0.36) |
|  | Heart rate and pulse investigations |  | 23(0.43) |
|  | Imaging procedures NEC |  | 57(1.07) |
|  | Investigations NEC |  | 53(0.99) |
|  | Microbiology and serology tests NEC |  | 17(0.32) |
|  | Mineral and electrolyte analyses |  | 13(0.24) |
|  | Neurologic diagnostic procedures |  | 22(0.41) |
|  | Physical examination procedures and organ system status |  | 38(0.71) |
|  | Respiratory tract and thoracic imaging procedures |  | 31(0.58) |
|  | Vascular tests NEC (incl blood pressure) |  | 28(0.52) |
| Metabolism and nutrition disorders | Appetite disorders | 22(0.41) | 22(0.41) |
| Musculoskeletal and connective tissue disorders | Joint related signs and symptoms | 651(12.19) | 97(1.82) |
|  | Muscle pains |  | 59(1.11) |
|  | Muscle related signs and symptoms NEC |  | 21(0.39) |
|  | Muscle weakness conditions |  | 29(0.54) |
|  | Musculoskeletal and connective tissue conditions NEC |  | 88(1.65) |
|  | Musculoskeletal and connective tissue pain and discomfort |  | 357(6.68) |
| Nervous system disorders | Acute polyneuropathies | 470(8.80) | 16(0.30) |
|  | Coordination and balance disturbances |  | 22(0.41) |
|  | Disturbances in consciousness NEC |  | 39(0.73) |
|  | Facial cranial nerve disorders |  | 10(0.19) |
|  | Headaches NEC |  | 146(2.73) |
|  | Neurological signs and symptoms NEC |  | 97(1.82) |
|  | Paraesthesias and dysaesthesias |  | 93(1.74) |
|  | Paralysis and paresis (excl cranial nerve) |  | 12(0.22) |
|  | Tremor (excl congenital) |  | 35(0.66) |
| Psychiatric disorders | Confusion and disorientation | 34(0.63) | 14(0.26) |
|  | Disturbances in initiating and maintaining sleep |  | 20(0.37) |
| Respiratory, thoracic and mediastinal disorders | Breathing abnormalities | 226(4.23) | 67(1.25) |
|  | Bronchospasm and obstruction |  | 10(0.19) |
|  | Coughing and associated symptoms |  | 65(1.22) |
|  | Pharyngeal disorders (excl infections and neoplasms) |  | 10(0.19) |
|  | Upper respiratory tract signs and symptoms |  | 74(1.38) |
| Skin and subcutaneous tissue disorders | Apocrine and eccrine gland disorders | 584(10.93) | 39(0.73) |
|  | Bullous conditions |  | 10(0.19) |
|  | Dermal and epidermal conditions NEC |  | 69(1.28) |
|  | Erythemas |  | 182(3.41) |
|  | Pruritus NEC |  | 80(1.50) |
|  | Rashes, eruptions and exanthems NEC |  | 160(3.00) |
|  | Urticarias |  | 44(0.82) |
| Social circumstances | Disability issues | 53(0.99) | 53(0.99) |
| Surgical and medical procedures | Therapeutic procedures NEC | 15(0.28) | 15(0.28) |
| Vascular disorders | Peripheral vascular disorders NEC | 16(0.30) | 16(0.30) |
| **Total number** | **75** | **5342(100.00)** | |

**Table S5.** **Adverse event following immunization (AEFI) associated with delayed recovery in the hypertensive population after trivalent influenza vaccines (TIA) vaccination.**

| **SOC** | **HLT** | | **No recovery group** | | **HR** | **95%CI** |
| --- | --- | --- | --- | --- | --- | --- |
|  |  |  | **Total (%)** | **N (%)** |  |  |
| General disorders and administration site conditions | Injection site reactions | | 1402(61.68) | 1070(47.07) | 2.04 | 1.22–3.40 |
|  | Pain and discomfort NEC | |  | 298(13.11) | 1.99 | 1.17–3.40 |
|  | Therapeutic and nontherapeutic responses | |  | 34(1.50) | 3.91 | 2.02–7.60 |
| Injury, poisoning and procedural complications | Skin injuries NEC | | 37(1.63) | 37(1.63) | 2.12 | 1.08–4.20 |
| Investigations | Imaging procedures NEC | | 57(2.51) | 57(2.51) | 1.82 | 1.02–3.30 |
| Musculoskeletal and connective tissue disorders | Joint related signs and symptoms | | 542(23.85) | 97(4.27) | 2.40 | 1.38–4.20 |
|  | Musculoskeletal and connective tissue conditions NEC | |  | 88(3.87) | 2.34 | 1.33–4.10 |
|  | Musculoskeletal and connective tissue pain and discomfort | |  | 357(15.71) | 2.40 | 1.43–4.10 |
| Nervous system disorders | Paraesthesias and dysaesthesias | | 93(4.09) | 93(4.09) | 2.34 | 1.34–4.10 |
| Psychiatric disorders | Disturbances in initiating and maintaining sleep | | 20(0.88) | 20(0.88) | 2.74 | 1.34–5.60 |
| Skin and subcutaneous tissue disorders | Dermal and epidermal conditions NEC | | 69(3.04) | 69(3.04) | 1.91 | 1.05–3.50 |
| Social circumstances | Disability issues | | 53(2.33) | 53(2.33) | 2.19 | 1.20–4.00 |
| **Total number** | | **12** | **2273(100.00)** | | | |

SOC, systematic organ classification; HLT, high-level terms; HR, hazard ratios; CI, confidence intervals.

**Table S6. Adverse events (AEs) with quadrivalent influenza vaccines (QIA) vaccination in the PS-matched hypertensive population.**

| **SOC** | **HLT** | **Total (%)** | **N (%)** |
| --- | --- | --- | --- |
| Blood and lymphatic system disorders | Lymphatic system disorders NEC | 24(0.37) | 24(0.37) |
| Cardiac disorders | Cardiac signs and symptoms NEC | 12(0.18) | 12(0.18) |
| Ear and labyrinth disorders | Ear disorders NEC | 46(0.70) | 12(0.18) |
|  | Hearing losses |  | 17(0.26) |
|  | Inner ear signs and symptoms |  | 17(0.26) |
| Eye disorders | Ocular disorders NEC | 75(1.15) | 29(0.44) |
|  | Ocular infections, inflammations and associated manifestations |  | 28(0.43) |
|  | Visual disorders NEC |  | 18(0.28) |
| Gastrointestinal disorders | Diarrhoea (excl infective) | 330(5.05) | 53(0.81) |
|  | Gastrointestinal and abdominal pains (excl oral and throat) |  | 27(0.41) |
|  | Gastrointestinal signs and symptoms NEC |  | 36(0.55) |
|  | Nausea and vomiting symptoms |  | 164(2.51) |
|  | Oral soft tissue signs and symptoms |  | 32(0.49) |
|  | Oral soft tissue swelling and oedema |  | 18(0.28) |
| General disorders and administration site conditions | Adverse effect absent | 2642(40.47) | 25(0.38) |
|  | Asthenic conditions |  | 298(4.56) |
|  | Febrile disorders |  | 148(2.27) |
|  | Feelings and sensations NEC |  | 267(4.09) |
|  | Gait disturbances |  | 39(0.60) |
|  | General signs and symptoms NEC |  | 278(4.26) |
|  | Inflammations |  | 20(0.31) |
|  | Injection site reactions |  | 1105(16.92) |
|  | Oedema NEC |  | 13(0.20) |
|  | Pain and discomfort NEC |  | 350(5.36) |
|  | Therapeutic and nontherapeutic responses |  | 64(0.98) |
|  | Vaccination site reactions |  | 35(0.54) |
| Immune system disorders | Allergic conditions NEC | 18(0.28) | 18(0.28) |
| Infections and infestations | Bacterial infections NEC | 67(1.03) | 41(0.63) |
|  | Herpes viral infections |  | 13(0.20) |
|  | Influenza viral infections |  | 13(0.20) |
| Injury, poisoning and procedural complications | Non-site specific injuries NEC | 128(1.96) | 21(0.32) |
|  | Product administration errors and issues |  | 78(1.20) |
|  | Skin injuries NEC |  | 19(0.29) |
|  | Vaccination related complications |  | 10(0.15) |
| Investigations | Autoimmunity analyses | 558(8.55) | 12(0.18) |
|  | Blood counts NEC |  | 24(0.37) |
|  | Carbohydrate tolerance analyses (incl diabetes) |  | 18(0.28) |
|  | Cardiac function diagnostic procedures |  | 20(0.31) |
|  | Central nervous system imaging procedures |  | 25(0.38) |
|  | Cerebrospinal fluid tests (excl microbiology) |  | 12(0.18) |
|  | ECG investigations |  | 35(0.54) |
|  | Heart rate and pulse investigations |  | 40(0.61) |
|  | Hepatobiliary function diagnostic procedures |  | 11(0.17) |
|  | Imaging procedures NEC |  | 69(1.08) |
|  | Investigations NEC |  | 76(1.16) |
|  | Metabolism tests NEC |  | 11(0.17) |
|  | Mineral and electrolyte analyses |  | 10(0.15) |
|  | Musculoskeletal and soft tissue imaging procedures |  | 12(0.18) |
|  | Neurologic diagnostic procedures |  | 25(0.38) |
|  | Physical examination procedures and organ system status |  | 46(0.70) |
|  | Protein analyses NEC |  | 15(0.23) |
|  | Respiratory tract and thoracic imaging procedures |  | 30(0.46) |
|  | Vascular tests NEC (incl blood pressure) |  | 38(0.58) |
|  | Virus identification and serology |  | 19(0.29) |
|  | White blood cell analyses |  | 10(0.15) |
| Metabolism and nutrition disorders | Appetite disorders | 28(0.43) | 28(0.43) |
| Musculoskeletal and connective tissue disorders | Bone related signs and symptoms | 826(12.65) | 11(0.17) |
|  | Bursal disorders |  | 12(0.18) |
|  | Joint related disorders NEC |  | 16(0.25) |
|  | Joint related signs and symptoms |  | 150(2.30) |
|  | Muscle pains |  | 67(1.03) |
|  | Muscle related signs and symptoms NEC |  | 37(0.57) |
|  | Muscle weakness conditions |  | 38(0.58) |
|  | Musculoskeletal and connective tissue conditions NEC |  | 117(1.78) |
|  | Musculoskeletal and connective tissue pain and discomfort |  | 378(5.79) |
| Nervous system disorders | Acute polyneuropathies | 668(10.23) | 30(0.46) |
|  | Coordination and balance disturbances |  | 33(0.51) |
|  | Disturbances in consciousness NEC |  | 52(0.80) |
|  | Facial cranial nerve disorders |  | 23(0.35) |
|  | Headaches NEC |  | 184(2.82) |
|  | Neurological signs and symptoms NEC |  | 141(2.16) |
|  | Paraesthesias and dysaesthesias |  | 151(2.31) |
|  | Seizures and seizure disorders NEC |  | 10(0.15) |
|  | Sensory abnormalities NEC |  | 21(0.32) |
|  | Tremor (excl congenital) |  | 23(0.35) |
| Psychiatric disorders | Anxiety symptoms | 77(1.18) | 12(0.18) |
|  | Confusion and disorientation |  | 20(0.31) |
|  | Disturbances in initiating and maintaining sleep |  | 28(0.43) |
|  | Sleep disorders NEC |  | 17(0.26) |
| Respiratory, thoracic and mediastinal disorders | Breathing abnormalities | 210(3.22) | 80(1.24) |
|  | Bronchospasm and obstruction |  | 13(0.20) |
|  | Coughing and associated symptoms |  | 46(0.70) |
|  | Pharyngeal disorders (excl infections and neoplasms) |  | 14(0.21) |
|  | Upper respiratory tract signs and symptoms |  | 57(0.87) |
| Skin and subcutaneous tissue disorders | Apocrine and eccrine gland disorders | 735(11.26) | 53(0.81) |
|  | Bullous conditions |  | 10(0.15) |
|  | Dermal and epidermal conditions NEC |  | 91(1.39) |
|  | Erythemas |  | 188(2.88) |
|  | Pruritus NEC |  | 139(2.13) |
|  | Rashes, eruptions and exanthems NEC |  | 182(2.80) |
|  | Urticarias |  | 72(1.10) |
| Social circumstances | Disability issues | 39(0.60) | 39(0.60) |
| Surgical and medical procedures | Therapeutic procedures NEC | 20(0.31) | 20(0.31) |
| Vascular disorders | Peripheral vascular disorders NEC | 26(0.40) | 26(0.40) |
| **Total number** | **94** | **6529(100.00)** | |

**Table S7. Adverse event following immunization (AEFI) associated with delayed recovery in the hypertensive population after** **quadrivalent influenza vaccines (QIA) vaccination.**

| **SOC** | **HLT** | **No recovery group** | | **HR** | **95%CI** |
| --- | --- | --- | --- | --- | --- |
|  |  | **Total (%)** | **N (%)** |  |  |
| Ear and labyrinth disorders | Ear disorders NEC | 46(0.83) | 12(0.22) | 4.06 | 1.85–8.89 |
|  | Hearing losses |  | 17(0.31) | 5.35 | 2.74–10.43 |
|  | Inner ear signs and symptoms |  | 17(0.31) | 2.58 | 1.14–5.84 |
| Eye disorders | Ocular disorders NEC | 57(1.03) | 29(0.52) | 2.60 | 1.30–5.21 |
|  | Ocular infections, inflammations and associated manifestations |  | 28(0.51) | 3.49 | 1.77–6.88 |
| Gastrointestinal disorders | Gastrointestinal and abdominal pains  (excl oral and throat) | 227(4.11) | 27(0.49) | 2.23 | 1.11–4.47 |
|  | Gastrointestinal signs and symptoms NEC |  | 36(0.65) | 2.09 | 1.04–4.18 |
|  | Nausea and vomiting symptoms |  | 164(2.97) | 2.20 | 1.33–3.66 |
| General disorders and administration site conditions | Asthenic conditions | 2582(46.74) | 298(5.39) | 2.25 | 1.41–3.58 |
|  | Febrile disorders |  | 148(2.68) | 2.26 | 1.35–3.76 |
|  | Feelings and sensations NEC |  | 267(4.83) | 2.12 | 1.31–3.43 |
|  | Gait disturbances |  | 39(0.71) | 1.90 | 1.05–3.45 |
|  | General signs and symptoms NEC |  | 278(5.03) | 2.31 | 1.45–3.69 |
|  | Inflammations |  | 20(0.36) | 4.53 | 2.29–8.95 |
|  | Injection site reactions |  | 1105(20.00) | 2.81 | 1.81–4.37 |
|  | Oedema NEC |  | 13(0.24) | 3.79 | 1.67–8.58 |
|  | Pain and discomfort NEC |  | 350(6.34) | 3.67 | 2.33–5.78 |
|  | Therapeutic and nontherapeutic responses |  | 64(1.16) | 2.87 | 1.65–4.99 |
| Infections and infestations | Influenza viral infections | 13(0.24) | 13(0.24) | 2.44 | 1.08–5.53 |
| Injury, poisoning and procedural complications | Skin injuries NEC | 29(0.52) | 19(0.34) | 5.85 | 2.92–11.74 |
|  | Vaccination related complications |  | 10(0.18) | 3.39 | 1.50–7.68 |
| Investigations | Blood counts NEC | 330(5.97) | 24(0.43) | 2.22 | 1.11–4.45 |
|  | Carbohydrate tolerance analyses (incl diabetes) |  | 18(0.33) | 3.18 | 1.40–7.21 |
|  | Heart rate and pulse investigations |  | 40(0.72) | 2.18 | 1.05–4.54 |
|  | Imaging procedures NEC |  | 69(1.25) | 2.43 | 1.47–4.02 |
|  | Investigations NEC |  | 76(1.38) | 1.88 | 1.12–3.15 |
|  | Musculoskeletal and soft tissue imaging procedures |  | 12(0.22) | 2.75 | 1.26–6.03 |
|  | Physical examination procedures and organ system status |  | 46(0.83) | 1.97 | 1.09–3.57 |
|  | Protein analyses NEC |  | 15(0.27) | 2.80 | 1.37–5.717 |
|  | Respiratory tract and thoracic imaging procedures |  | 30(0.54) | 2.08 | 1.11–3.92 |
| Metabolism and nutrition disorders | Appetite disorders | 28(0.51) | 28(0.51) | 2.57 | 1.30–5.07 |
| Musculoskeletal and connective tissue disorders | Bone related signs and symptoms | 826(14.95) | 11(0.20) | 4.02 | 1.70–9.48 |
|  | Bursal disorders |  | 12(0.22) | 5.57 | 2.73–11.36 |
|  | Joint related disorders NEC |  | 16(0.29) | 2.32 | 1.17–4.57 |
|  | Joint related signs and symptoms |  | 150(2.72) | 3.33 | 2.08–5.34 |
|  | Muscle pains |  | 67(1.21) | 3.13 | 1.84–5.33 |
|  | Muscle related signs and symptoms NEC |  | 37(0.67) | 2.11 | 1.11–4.02 |
|  | Muscle weakness conditions |  | 38(0.69) | 2.51 | 1.43–4.40 |
|  | Musculoskeletal and connective tissue conditions NEC |  | 117(2.12) | 3.74 | 2.31–6.06 |
|  | Musculoskeletal and connective tissue pain and discomfort |  | 378(6.84) | 3.69 | 2.35–5.78 |
| Nervous system disorders | Coordination and balance disturbances | 532(9.63) | 33(0.60) | 2.24 | 1.18–4.26 |
|  | Facial cranial nerve disorders |  | 23(0.42) | 2.38 | 1.29–4.41 |
|  | Headaches NEC |  | 184(3.33) | 2.25 | 1.38–3.67 |
|  | Neurological signs and symptoms NEC |  | 141(2.55) | 1.85 | 1.07–3.20 |
|  | Paraesthesias and dysaesthesias |  | 151(2.73) | 3.30 | 2.06–5.29 |
| Psychiatric disorders | Anxiety symptoms | 77(1.39) | 12(0.22) | 3.20 | 1.20–8.52 |
|  | Confusion and disorientation |  | 20(0.36) | 4.89 | 2.44–9.81 |
|  | Disturbances in initiating and maintaining sleep |  | 28(0.51) | 4.50 | 2.41–8.41 |
|  | Sleep disorders NEC |  | 17(0.31) | 3.63 | 1.78–7.41 |
| Respiratory, thoracic and mediastinal disorders | Coughing and associated symptoms | 46(0.83) | 46(0.83) | 2.05 | 1.09–3.86 |
| Skin and subcutaneous tissue disorders | Dermal and epidermal conditions NEC | 672(12.17) | 91(1.65) | 2.48 | 1.48–4.15 |
|  | Erythemas |  | 188(3.40) | 2.31 | 1.43–3.74 |
|  | Pruritus NEC |  | 139(2.52) | 2.58 | 1.56–4.25 |
|  | Rashes, eruptions and exanthems NEC |  | 182(3.29) | 2.08 | 1.28–3.38 |
|  | Urticarias |  | 72(1.30) | 2.10 | 1.16–3.79 |
| Social circumstances | Disability issues | 39(0.71) | 39(0.71) | 2.84 | 1.57–5.12 |
| Surgical and medical procedures | Therapeutic procedures NEC | 20(0.36) | 20(0.36) | 2.65 | 1.32–5.32 |
| **Total number** | **57** | **5524(100.00)** | | | |

SOC, systematic organ classification; HLT, high-level terms; HR, hazard ratios; CI, confidence intervals.

**Table S8. Adverse events (AEs) with quadrivalent influenza vaccines (QIA) vaccination in the PS-matched hypertensive population.**

| **SOC** | **HLT** | **Total (%)** | **N (%)** |
| --- | --- | --- | --- |
| Eye disorders | Ocular infections, inflammations and associated manifestations | 5(1.22) | 5(1.22) |
| Gastrointestinal disorders | Nausea and vomiting symptoms | 10(2.44) | 10(2.44) |
| General disorders and administration site conditions | Asthenic conditions | 199(48.54) | 23(5.61) |
|  | Febrile disorders |  | 14(3.41) |
|  | Feelings and sensations NEC |  | 23(5.61) |
|  | Gait disturbances |  | 6(1.46) |
|  | General signs and symptoms NEC |  | 20(4.88) |
|  | Injection site reactions |  | 83(20.25) |
|  | Pain and discomfort NEC |  | 25(6.10) |
|  | Therapeutic and nontherapeutic responses |  | 5(1.22) |
| Investigations | Imaging procedures NEC | 14(3.41) | 6(1.46) |
|  | Investigations NEC |  | 8(1.95) |
| Musculoskeletal and connective tissue disorders | Joint related signs and symptoms | 73(17.80) | 13(3.17) |
|  | Muscle pains |  | 6(1.46) |
|  | Musculoskeletal and connective tissue conditions NEC |  | 16(3.90) |
|  | Musculoskeletal and connective tissue pain and discomfort |  | 38(9.27) |
| Nervous system disorders | Headaches NEC | 42(10.24) | 17(4.14) |
|  | Neurological signs and symptoms NEC |  | 10(2.44) |
|  | Paraesthesias and dysaesthesias |  | 15(3.66) |
| Psychiatric disorders | Disturbances in initiating and maintaining sleep | 5(1.22) | 5(1.22) |
| Respiratory, thoracic and mediastinal disorders | Breathing abnormalities | 26(6.35) | 7(1.71) |
|  | Coughing and associated symptoms |  | 10(2.44) |
|  | Upper respiratory tract signs and symptoms |  | 9(2.20) |
| Skin and subcutaneous tissue disorders | Dermal and epidermal conditions NEC | 30(7.32) | 5(1.22) |
|  | Erythemas |  | 12(2.93) |
|  | Pruritus NEC |  | 7(1.71) |
|  | Rashes, eruptions and exanthems NEC |  | 6(1.46) |
| Social circumstances | Disability issues | 6(1.46) | 6(1.46) |
| **Total number** | **28** | **410(100.00)** | |

**Table S9. Adverse event following immunization (AEFI) associated with delayed recovery in the hypertensive population after influenza unknown manufacturer (FLUX) vaccination.**

| **SOC** | **HLT** | **No recovery group** | | **HR** | **95%CI** |
| --- | --- | --- | --- | --- | --- |
|  |  | **Total (%)** | **N (%)** |  |  |
| General disorders and administration site conditions | Injection site reactions | 88(71.54) | 83(67.48) | 2.83 | 1.12–7.15 |
|  | Therapeutic and nontherapeutic responses |  | 5(4.07) | 6.31 | 1.50–26.53 |
| Investigations | Imaging procedures NEC | 6(4.88) | 6(4.88) | 4.20 | 1.21–14.56 |
| Musculoskeletal and connective tissue disorders | Joint related signs and symptoms | 29(23.58) | 13(10.57) | 3.75 | 1.25–11.26 |
|  | Musculoskeletal and connective tissue conditions NEC |  | 16(13.01) | 3.64 | 1.29–10.23 |
| **Total number** | **5** | **123(100.00)** | | | |

SOC, systematic organ classification; HLT, high-level terms; HR, hazard ratios; CI, confidence intervals.

**Fig S1. Data collection and design.**


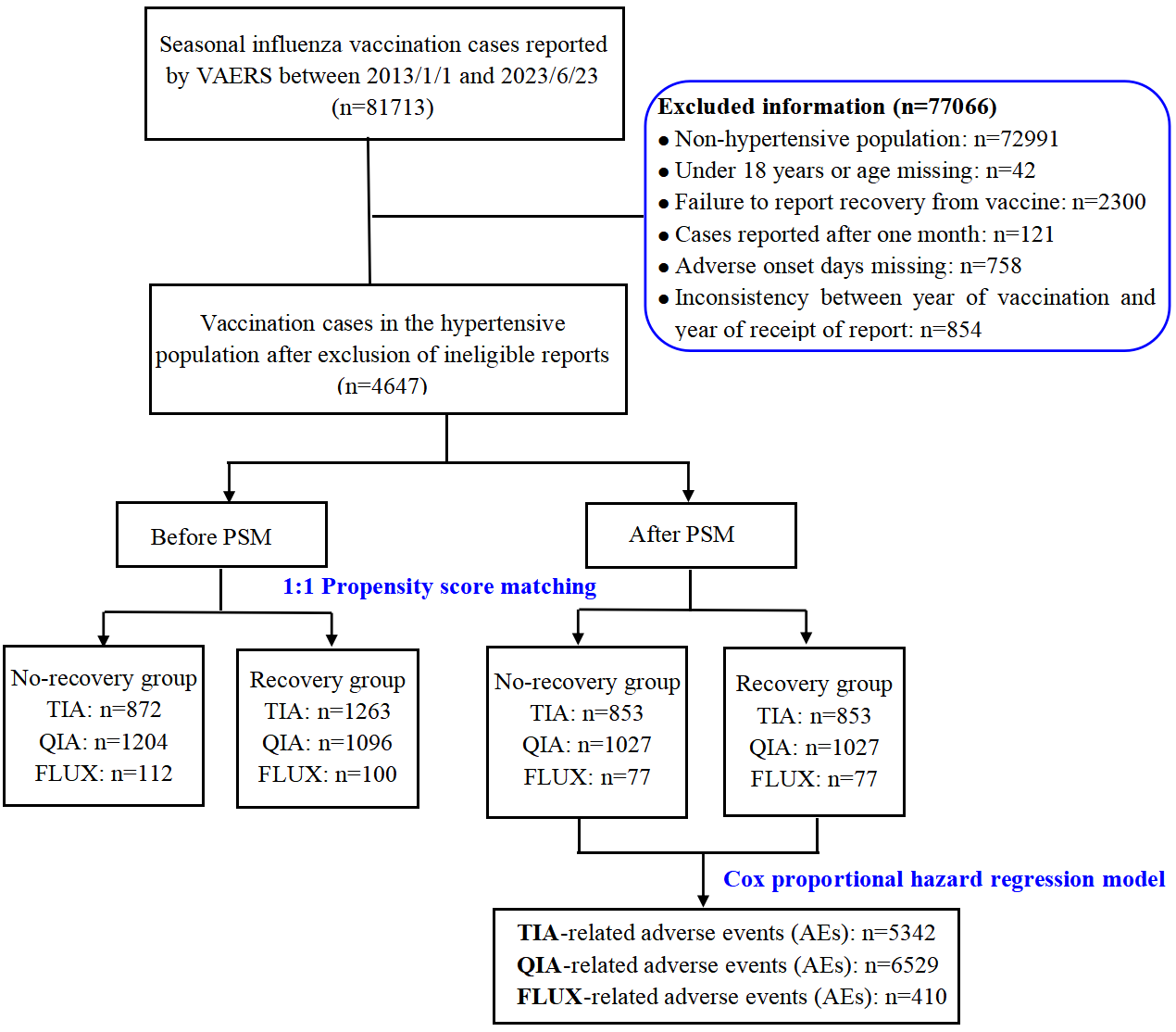


**Fig S2. Kaplan-Meier plots comparing the cumulative incidence of non-recovery in the hypertensive population following three types of seasonal influenza vaccines within one month.**


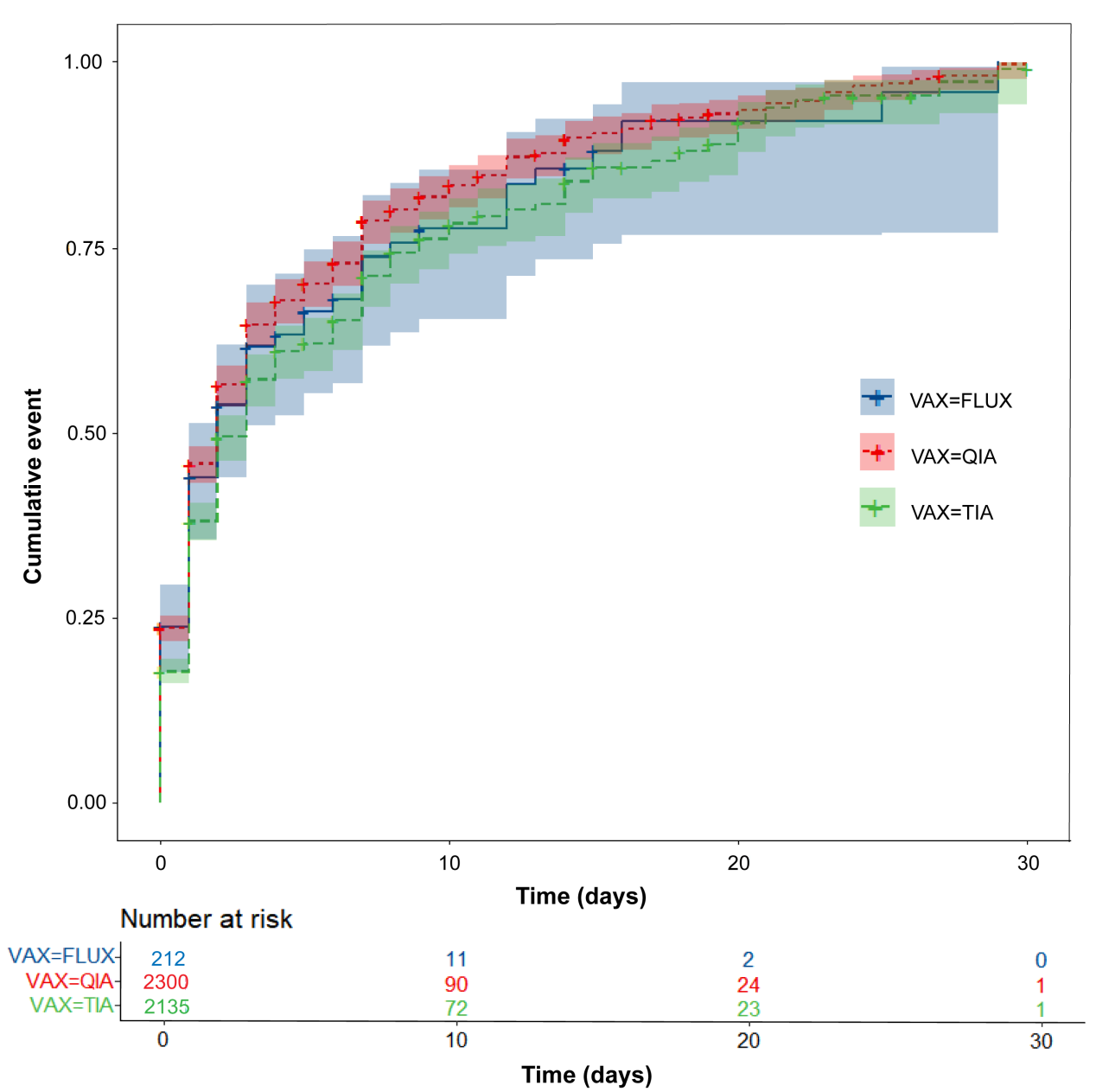


**Fig S3.** **Comparison of propensity score matching (PSM) before and after trivalent influenza vaccines (TIA), quadrivalent influenza vaccines (QIA) and influenza unknown manufacturer (FLUX) vaccinations.**


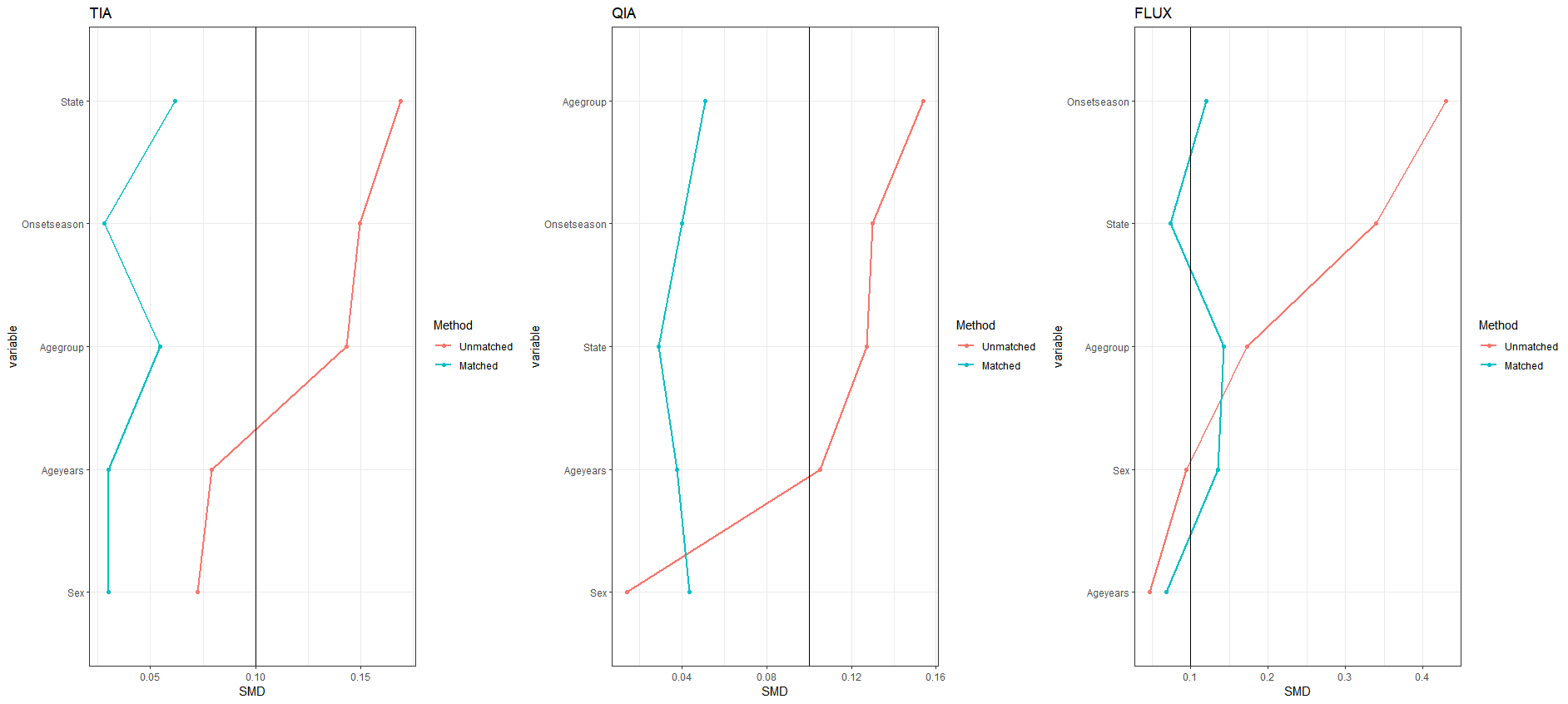

Supplement: S1 Appendix — (DOCX) [file pone.0310474.s001.docx]
